# Intelligent Biosignal Processing in Wearable and Implantable Sensors

**DOI:** 10.3390/bios12060396

**Published:** 2022-06-09

**Authors:** Hariton-Nicolae Costin, Saeid Sanei

**Affiliations:** 1 Institute of Computer Science, Romanian Academy, 700481 Iasi, Romania; 2 School of Science and Technology, Nottingham Trent University, Nottingham NG11 8NS, UK; saeid.sanei@ntu.ac.uk

## 1. Introduction

Wearable technology including sensors, sensor networks, and the associated devices have opened up space in a variety of applications. Long-term, noninvasive, and nonintrusive monitoring of the human body through collecting as many biometrics and body state indicators as possible is the major goal of healthcare wearable technology developers. Patients suffering diabetes need a simple noninvasive tool to monitor their blood sugar on an hourly basis. Those suffering from seizures require the necessary instrumentation to alarm them before any seizure onset to prevent them from a fall injury. Stroke patients need their heart rate recorded constantly. These are only some examples to show how crucial and necessary the wearable healthcare systems can be.

A remote low-cost monitoring strategy significantly promotes social and clinical wellbeing. This can only be achieved if sufficiently reliable recorded information from the human body is available. Such information may be metabolic, biological, physiological, behavioural, psychological, functional, or movement-related.

On the other hand, the development of mobile telephones since the early 1990’s and their improvement till now, together with the availability of large size memory and wideband communication channels, make it significantly easier to achieve the above objectives without hospitalising patients in hospitals and care units for a long time. This may be considered a revolution in human welfare. Therefore, more effective and efficient collection of biosignals and biometrics from the human body has a tremendous impact and influence on healthcare and the technology involved.

The state of a patient during rest, walking, working, and sleeping can be well recognised if all the biomarkers of the physiological, biological, and behavioural changes of human body can be measured and processed. This requirement sparks the need for the deployment of wearable multi-sensor and multimodal data collection systems. Hence, wearable technology and body sensor networks are central to a complete solution for patient monitoring and healthcare.

The measurable underlying information, however, may not be always visualized by the naked eye, and therefore, signal processing, machine learning, and artificial intelligence (AI) techniques have been constantly under research and development in the hope that these techniques can achieve a better understanding and recognition of the human body state from raw data records. Although the objective is to have noninvasive and less intrusive sensors, the use of implanted sensors becomes inevitable for particular in-vivo recordings where the human bioindicators need to be monitored for a longer time.

The incorporation of AI into medical care leads to the so-called third generation of pervasive health applications. This recent branch of research area aims to combine continuous health monitoring with other sources of medical information and knowledge. Thus, the main objective in third-generation applications is to integrate intelligent agents that implement technologies such as stream and real-time processing, data mining, machine learning, and genetic and multi-omics data. On the other hand, the use of smart sensors paves the path for personalized medicine, which is one of the objectives of future healthcare. With more intelligent systems developed through advanced processing and learning algorithms, the number of sensors can also be reduced, as less intrusive monitoring is another objective.

This Special Issue aims to address major advances in the integration and intelligent processing of data coming from wearable, portable, or implantable clinically approved devices. It is also intended to highlight new research opportunities in biomedical informatics and the clinical environment. The incorporation of on-chip machine learning and AI can lead to the realization of smart sensors.

Delightfully, this Special Issue attracted the attention of a large number of authors enthusiastically working in the related areas. Among them the following submission topics successfully achieved the goals of this issue due to their pioneering contributions in the field.

The study by Sawan et al. [1] applies EEG-based brain-machine interfaces during medical rehabilitation, by separating various tasks during motor imagery (MI) and assimilating MI into motor execution (ME). The authors implement intelligent, straightforward, comprehensible, time-efficient, and channel-reduced methods to classify ME versus MI and left- versus right-hand MI. Aside from time-domain information, they map EEG signals to feature space, using extraction methods including statistics, wavelet coefficients, average power, sample entropy, and common spatial patterns. To evaluate their practicability, a support-vector machine as an intelligent classifier model and sparse logistic regression as a feature-selection technique were adopted, and a rate of 79.51% accuracy was obtained. The achieved results make the proposed approach highly suitable to be applied to the rehabilitation of paralyzed limbs.

The paper by Lee et al. [2] analyzes the misalignments and detection errors of quasi-synchronous alignment between echocardiography images and seismocardiogram signals, the latter coming from accelerometer-based devices. Two diagnostic parameters—the ratio of pre-ejection period to left ventricular ejection time (PEP/LVET) and the Tei index—were examined with two statistical verification approaches. In this context, a dynamic time warping (DTW) algorithm was used to align fiducial points. The proposed approach may enable the standardization of the fiducial point detection and the signal template generation. In this way, the program-generated annotation data may serve as the labeled training set for the supervised machine-learning instrument.

The paper by Liu et al. [3] is dedicated to the evaluation of sympathetic nerve activity (SNA), using a skin sympathetic nerve activity (SKNA) signal by means of a Teager-Kaiser energy (TKE) operator, which preprocesses the SKNA signal. The SKNA energy ratio (SKNAER) was proposed for quantifying the SKNA. SKNAER improved the detection accuracy for the burst of SKNA, with 98.2% for detection rate and 91.9% for precision, compared to other approaches. The authors appreciate that the proposed developed feature may play an important role in continuously monitoring of SNA and containing potential for further clinical tests.

COVID-19 could not be missing from this Special Issue. The study by Attallah et al. [4] introduces a novel automated diagnostic tool based on ECG data to diagnose COVID-19, which utilizes 10 deep learning (DL) models of various architectures. It obtains significant features from the last fully connected layer of each DL model and then combines them. Afterward, the tool presents a hybrid feature selection based on the chi-square test and sequential search to select significant features. Finally, it employs several machine-learning classifiers to perform two classification levels: a binary level to differentiate between normal and COVID-19 cases and a multiclass to discriminate COVID-19 cases from normal and other cardiac complications. The proposed method reached an accuracy of 98.2% and 91.6% for binary and multiclass levels, respectively. This performance indicates that the ECG could be used as an alternative means of diagnosis of COVID-19, and perhaps for other diseases.

The study by Rieta et al. [5] proposes a classification model to discriminate between normotensive and hypertensive subjects, employing electrocardiographic and photoplethysmographic (PPG) recordings as an alternative to traditional cuff-based methods. By using 17 discriminatory features extracted from the ECG signal, the main outcome of this research uncovers the relevance of previous calibration to obtain accurate hypertension risk assessment. The k-nearest neighbor classifier provided the best outcomes with an accuracy for new subjects before calibration of 51.48%. The inclusion of just one calibration measurement into the model improved classification accuracy by 30%, reaching gradually more than 96%. Thus, the use of PPG and ECG recordings combined with previous subject calibration can significantly improve discrimination between normotensive and hypertensive individuals.

The paper by Faragó et al. [6] proposes a wearable physiograph for qualitative and quantitative Parkinsonians gait assessment, which performs bilateral tracking of the foot biomechanics and unilateral tracking of arm balance. In this way, the main objective is the monitoring and assessment of gait in Parkinson’s disease patients. The novelty is given by the proposed AI-based decisional support procedure for gait assessment, which is validated in a clinical environment. The authors claim that a platform empowering multidisciplinary, AI-evidence-based decision support assessments for optimal dosing between drug and non-drug therapy could lay the foundation for affordable precision medicine.

In [7], the authors analyze the gait signal obtained from an inertial-sensor-based wearable gait device as a tool to manage bone loss and muscle loss in daily life and classify them into seven gait phases. Then, they use explainable AI to analyze the contribution and importance of descriptive statistical parameters on osteopenia and sarcopenia. They confirm high classification accuracy and the statistical significance of gait factors used for osteopenia and sarcopenia management

In [8], the authors propose a comparative analysis of the projection matrices and dictionaries used for compressive sensing (CS) of electrocardiographic (ECG) signals by making compromises between the complexity of preprocessing and the accuracy of reconstruction. They use several types of projection matrices and the reconstructed signals are analyzed quantitatively and qualitatively.

Roy et al. [9] developed an auto-characterization algorithm to leverage the AI-powered auto-signal-enhancing scheme such as denoising autoencoder and adaptive cell characterization technique based on the transfer of learning in deep neural networks. They reported a considerable increase in accuracy and signal enhancement.

In [10], the authors use a carbon nanotube yarn (CNTY) biosensor to chronically record from the vagus nerves of freely moving rats for over 40 continuous hours. Vagal activity is analyzed and spike-cluster-firing rates are found to correlate with food intake. Hence, the neural-firing rates are used to classify eating and other behaviors. This is claimed to be the first chronic recording and decoding of activity in the vagus nerve of freely moving animals enabled by the axon-like properties of the CNTY biosensor in both size and flexibility. This technology is an important step forward in understanding spontaneous vagus-nerve function.

The purpose of the exploratory study by Reuken et al. [11] is to determine whether liver dysfunction can be generally classified with a wearable electronic nose based on semiconductor metal oxide (MOx) gas sensors, and whether the extent of this dysfunction can be quantified. Three sensor modules with a total of nine different MOx layers are used to detect reducible, easily oxidizable, and highly oxidizable gases through non-invasive, rapid, and cost-effective analysis.

Jiang et al. [12] have analyzed surface Electromyography (sEMG) and used it for prosthesis control. They explore how the grasp classification accuracy changes during reaching and grasping, and they identify the period during which the grasp classification accuracy and detection are high. This period has been found suitable for early grasp classification with reduced delay. They also explore the training strategies for better grasp classification in real-time applications.

Chon et al. [13] present an automated arterial fibrillation (AF) prediction algorithm for critically ill sepsis patients, using electrocardiogram (ECG) signals. They extract features from 5-min ECG, using the traditional time, frequency, and nonlinear domain methods. Different classifiers are then used to classify the existing cardiology dataset. The proposed algorithm achieved 80% accuracy for predicting AF events 10 min earlier than the AF onset.

Faupel et al. [14] use a convolutional neural network (CNN) for epileptic seizure detection capable of running on an ultra-low-power microprocessor, optimised and simulated by MATLAB and implemented on a GAP8 microprocessor with RISC-V architecture. It is claimed that the proposed detector outperforms related approaches in terms of power consumption by a factor of 6. The universal applicability of the proposed CNN based detector is verified with the recording of epileptic rats.

For classification of ECG and EEG signals, Goraș et al. [15] investigate three techniques for reducing dimensionality, namely Laplacian eigenmaps, locality preserving projections, and compressed sensing. The effect of dimensionality reduction is assessed by considering the classification rates for the processed biosignals in the new spaces with different classifiers.

An approach to detect premature ventricular contractions (PVCs) from long-term ECG has been proposed in [16]. The suggested method utilizes deep metric learning to extract features, with compact intra-product variance and separated inter-product differences, from the heartbeat. The use of k-nearest neighbor (KNN), together with the proposed feature extraction method, can extract features by supervised deep-metric learning, which can avoid the bias caused by manual feature engineering. The simulation events show that it is reliable to use deep metric learning and KNN for PVC recognition.

It is our great pleasure to invite you to read this diverse range of papers, as we are hopeful that these submitted works will constitute strong foundations for more research and development in the areas of sensors, wearable technology, and the related signal and data processing techniques by means of AI methods.
